# Evaluation of Vitamin D and of Some Biomarkers of Bone Remodelling (CTX-1, Osteocalcin, BALP) in Subjects with Periapical Inflammatory Cysts: An Observational Study

**DOI:** 10.3390/jcm14113712

**Published:** 2025-05-26

**Authors:** Angela Pia Cazzolla, Vincenzo Brescia, Roberto Lovero, Roberta Cardinali, Francesca Di Serio, Mauro Lorusso, Domenico Ciavarella, Nunzio Francesco Testa, Gianna Dipalma, Michele Di Cosola, Lorenzo Lo Muzio, Vito Crincoli, Mariasevera Di Comite

**Affiliations:** 1 Department of Clinical and Experimental Medicine, Università degli Studi di Foggia, 71122 Foggia, Italy; 2 Clinical Pathology Unit, AOU Policlinico Consorziale di Bari-Ospedale Giovanni XXIII, 70124 Bari, Italy; 3 Clinical Pathology and Neonatal Screening, Azienda Ospedaliera Universitaria Policlinico-Giovanni XXIII, 70124 Bari, Italy; 4 Interdisciplinary Department of Medicine, University of Bari Aldo Moro, Piazza G. Cesare 11, 70124 Bari, Italy; 5 Department of Translational Biomedicine and Neuroscience (DiBraiN), University of Bari “Aldo Moro”, Piazza Giulio Cesare, 70124 Bari, Italy

**Keywords:** periapical cysts, periapical diseases, bone metabolism, serum biomarkers, ROC curve analysis

## Abstract

**Objectives:** The aim of this observational study was to evaluate whether the presence of periapical inflammatory cysts (PIC) is accompanied by a state of vitamin D (25OHD) 25(OH)D insufficiency or deficiency and biochemical variations in biomarkers of bone metabolism such as osteocalcin (OC), isoenzyme of bone alkaline phosphatase (BALP), and C-terminal telopeptide of type 1 collagen (CTX). **Methods:** A total of 56 patients (group P), 36 males and 20 females, of which 42 had one cyst (group P1) and 14 had multiple periapical cysts (group P2), alongside 56 healthy subjects (group H) were recruited. Rx-OPT and clinical evaluation were used to evaluate the presence of PIC. At the first visit, all subjects underwent venous sampling (group P and H) to measure bone biomarkers by the chemiluminescence method. The Mann–Whitney test was used to compare the different biomarkers in the H vs. P, H vs. P1, H vs. P2, and P1 vs. P2 groups. The Mann–Whitney test was used to compare biomarker levels between the study groups. ROC curves were used to search for the concentration of the different biomarkers in which the best sensitivity and specificity were found. **Results:** 25OHD and CTX showed a difference between H vs. P, H vs. P1, H vs. P2, and P1 vs. P1 groups (*p* < 0.05). The study of the ROC curves with a comparison between concentrations in the H vs. P group showed the best sensitivity and specificity for 25OHD at a concentration <19 ng/mL, highlighting a picture of 25OHD deficiency. **Conclusions:** The presence of apical cysts could be indicative of a vitamin D deficiency that should be appropriately treated. The findings suggest that vitamin D deficiency, given its role in bone metabolism and mineralisation, may contribute to a biological environment that favours the development or persistence of periapical cystic lesions.

## 1. Introduction

Periapical lesions are acute or chronic inflammatory diseases of the periapical tissues of the tooth, which arise as a consequence of diseases of the endodontic tissues of the tooth and can cause destruction of the periapical bone [1,2]. Teeth represent a direct route for the spread of infection due to the absence of epithelial barriers to infectious and/or inflammatory agents [1].

Acute or chronic inflammatory reaction can develop depending on the state of the infection in the root canal (necrotic or infected) and on the host defense mechanisms [2].

Elevated levels of prostaglandins and cytokines have been found to be associated with periapical lesions in both human and animal studies [3]. These mediators are produced locally in periapical lesions and perform a communication function in the cross-regulation of immune responses and in the spread and evolution of these inflammatory processes [4].

Periapical inflammatory cysts (PIC) are often associated with necrotic teeth or inappropriate endodontic treatments and originate from epithelial residues (Malassez remnants) present in apical granulomas stimulated by cytokines produced by inflammation [4].

PIC are more than half of odontogenic cysts, and appear between the ages of 20 and 60, both in males and females, with a predominantly anterior maxillary location (60%) followed by the posterior maxillary, posterior mandibular, and anterior mandibular portions, with dimensions ranging from a few millimetres to a few centimetres. They are equipped with a capsule of fibrous tissue, lined with non-keratinised multistratified squamous epithelium, which can undergo mucosal metaplasia and contain mesenchymal stem cells [5], eosinophilic hyaline epithelial bodies (Rushton), and cholesterol deposits [1,6].

The therapy of PIC is determined by the location of the cyst, the anatomical relationships with essential structures, the clinical situation, and the patient’s general health status. Surgical therapy includes enucleation and marsupialisation [7], while non-surgical therapies are endodontic treatment [8], decompression [9,10], the aspiration and irrigation technique [11], therapy using calcium hydroxide [12,13], lesion sterilisation and repair [14], the Apexum procedure [15,16], and the use of simvastatin and epigallocatechin [17].

It is hypothesised that the mechanism of PIC growth depends both on cellular activity stimulated by the release of inflammatory mediators and on the increase in osmotic pressure within the lumen of the cyst, which attracts fluids from the cavity. The osmotic gradient favours the expansion of the cyst and stimulates the production of cytokines and other factors that activate fibroblasts and lead to bone resorption. Activated fibroblasts release collagenase and prostaglandins and activate osteoclasts with subsequent bone resorption [18].

Several bone biomarkers have been studied for the evaluation of bone remodelling during both acute and chronic inflammatory processes of the stomatognathic system, whose production could be influenced by the release of cytokines [19]. The aim of this preliminary study was to evaluate whether the presence of periapical inflammatory cysts (PIC) is accompanied by biochemical variations in biomarkers of bone metabolism such as osteocalcin (OC), isoenzyme of bone alkaline phosphatase (BALP), C-terminal telopeptide of type 1 collagen (CTX), and a state of vitamin D insufficiency or deficiency (25OHD).

Serum osteocalcin (OC) was evaluated because it is considered a specific marker of bone formation when formation and resorption are uncoupled and a valid marker of bone turnover when resorption and formation are coupled. It could function as a negative regulator because it could be involved in the recruitment of osteoclasts to sites of newly formed bone. Osteocalcin (OC) or bone Gla-protein is a small calcium-binding protein of bone, and it is the most abundant noncollagenous protein of mineralised tissues. It is a protein of 49 amino acids with a molecular mass of approximately 6kd. It is synthesised by osteoblasts, odontoblasts, and hypertrophic chondrocytes, and it has an important role in bone resorption and mineralisation [4,20,21].

Alkaline phosphatase bone isoenzyme (BALP) is a membrane enzyme and nonspecific hydrolase present in all tissues, particularly in bones and the liver, bile duct, intestinal mucosa, and kidneys [22]. High levels of BALP are associated with the process of calcification and active bone remodelling [23,24]. At the dental level, it participates in the normal turnover of the periodontal ligament, in the formation and maintenance of root cement and in bone homeostasis [25]. Studies conducted on an experimental model of gingivitis have highlighted a significant correlation between BALP and pocket depth and between BALP and inflammation [25]. For this reason, BALP could act as an indicator of periodontal disease, as well as in the planning and monitoring of periodontal treatment.

C-terminal telopeptide of type 1 collagen (CTX) and in particular serum β-CTX is a marker of bone resorption, produced during cathepsin K-mediated bone degradation processes when insoluble type I collagen is cleaved into different fragments in the resorption compartment of osteoclasts. Cathepsin K is active in fibroblasts, macrophages, and gingival epithelial cells and may promote bone resorption processes [26]. Therefore, serum levels are significantly correlated with histomorphometric measurements of bone resorption [27].

Vitamin D (25OHD) is a secosteroid hormone which plays multiple roles in cellular physiology, bone metabolism, and immunomodulatory functions. It is an important regulator of calcium balance because it participates in the processes that regulate the exchange of calcium ions and phosphate ions between the skeleton and blood; promotes the synthesis of some proteins, including osteocalcin, as well as the activation of osteoclasts; and has a calcium-fixing action on the bones [28]. Vitamin D 25 OHD is important for craniofacial and tooth development and in the maintenance of good oral health. In fact, the evidence supports an important association between vitamin D and dental-oral health [29]. Inadequate concentrations of vitamin D have been associated with periodontal disease, tooth and alveolar bone loss, and salivary gland atrophy. 25OHD deficiency during periods of primary and permanent tooth formation is responsible for a metabolic insult to ameloblasts that causes enamel hypoplasia, dentin/enamel hypo calcification, and dental caries [30].

In addition, vitamin D 25OHD has important effects on the immune system, directly inducing antimicrobial peptides on mucosal surfaces and modulating the function of T lymphocytes. In fact, it allows the activation of T lymphocytes by binding to the specific receptor, known as the 25OHD vitamin D receptor [31].

When a T cell is exposed to a pathogen, it exposes a signalling device known as a vitamin D receptor which, by binding to vitamin D, allows the activation of the T cell. In particular, it inhibits the production of cytokines by T-helper 1 (Th1) lymphocytes that trigger local inflammatory reactions and induce tissue injury, and it selectively induces the production of cytokines by T-helper 2 (Th2), which promote reparative responses and facilitate periodontal repair [3,32].

In fact, the specific suppression of Th1 cells inhibits alveolar bone loss in experimental animal models. Several observational studies have demonstrated a protective effect of 25OHD vitamin D against infections, and its deficiency facilitates the onset of infections and the evolution of inflammatory processes such as periodontal disease [33]. A recent European consensus stated that periodontal status and oral health are affected by poor nutrition and especially by inadequate 25OHD intake. In particular, low serum 25OHD levels are linked to increased severity of periodontitis and worse response to treatment. Therefore, 25OHD vitamin D plays a role in maintaining periodontal health and adequate bone mineral density [3].

## 2. Materials and Methods

### 2.1. Study Design and Participants

This observational study was conducted from January 2021 to June 2023, in accordance with the provisions of the Declaration of Helsinki. The study was approved by the Ethics Committee of Bari (Italy) (N. 6481 VIT.D-BALP INCLUSION protocol number 0054625/31 July 2020), and informed consent was obtained from each patient.

Fifty-six patients (33 men and 23 women; age between 21 and 56 years, mean age 33) affected by periapical odontogenic cysts (group P) and fifty-six subjects (35 men and 21 women) aged over 18 years (age between 20 and 56 years, mean age 30), apparently healthy (group H), were included in the study. Healthy subjects (group H) were recruited from a study group of patients who were to undergo orthodontic treatment.

### 2.2. Inclusion Criteria

**Figure 1:**
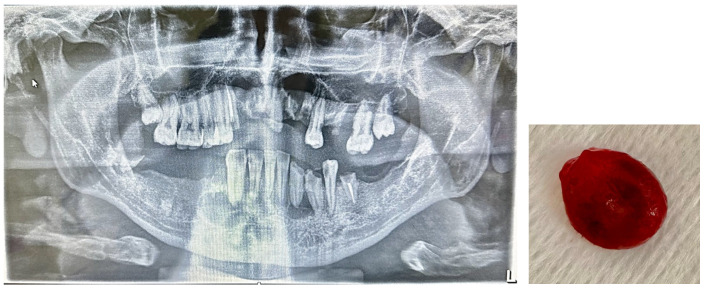
Presence of PIC in the 3.3 region in a patient with multiple cysts and root residues. The lesions were surgically removed (cystectomy with extraction of root residues).

The patients recruited they were non-smokers; had not undergone previous maxillofacial surgery; and presented at the complete dental examination the absence of active caries processes, gingival bleeding, or oral mucosal lesions. These patients did not present radiographic signs of periodontal disease and PIC at routine RX-OPT. The 56 patients (group P) presented radiographic signs of PIC such as homogeneous, well-defined radiolucent areas with clear margins; sclerotic rim, exceeding 5 mm in size; displacement of the roots of adjacent teeth; and/or root resorption associated with cortical expansion and thinning (Figure 1).

Of the 56 patients (group P), 42 (75%) had a single lesion (subgroup P1) and 14 (25%) had multiple lesions (subgroup P2). All Rx-OPT were reported by the same expert operator. In addition, all apparently healthy subjects (group H) and those with PIC (group P) had serum C-reactive protein less than 4 mg/L, serum creatinine range 0.6–1.17 mg/Dl, calcium range 8.5–10.6 mg/dL, and serum phosphate range 3.1–5.90 mg/dL.

### 2.3. Exclusion Criteria

Exclusion criteria were taking vitamin D replacement therapy, various cancers, known endocrine/metabolic diseases, hypophosphatemia, hypocalcaemia, renal insufficiency (eGFR < 75 mL/min/1.73 m^2^), hospitalisation in the last 4 months, bone fractures (≤3 months), and pregnancy.

### 2.4. Sample Management

A venous blood sample was collected from healthy subjects and each patient to measure vitamin D, BALP, CTX, and OC. Blood from the forearm vein was collected in 5 mL Vacutainer tubes without anticoagulant. Blood samples were centrifuged (1000× *g*, 15 min, 4 °C), and serum was removed and immediately stored at −80 °C until analysis. All samples were analysed within two months of storage.

### 2.5. Analytical Determination

The levels of BALP (ug/L), CTX (ng/mL), OC (ng/mL), and 25OHD 25(OH)D (ng/mL) in serum was measured by the chemiluminescence method on the TGSTA Technogenetics analyser (Technogenetics, Milan, Italy), using the IDS kits, iSYS Multi-Discipline Automated System (IDS, New Delhi, India). The method uses two monoclonal antibodies specific for the molecules to be measured linked to biotin and acridinium. The luminescence emitted by the acridinium labelling is directly proportional to the concentration of the biomarker in the sample under examination. The serum determinations of BALP (ug/L), CTX (ng/mL), OC (ng/mL), and 25OHD 25(OH)D (ng/mL) in serum followed the indications of the supplier company. In particular, preventive maintenance activities, possible calibrations, and analytical quality controls were carried out. In order to monitor analytical precision (CV%), materials for internal quality control (low, intermediate, and high level) provided by the manufacturing company were used; for analytical accuracy, the results of participation in EQAS (External Quality Assessment Services) programs were evaluated. The performances obtained were suitable for the analytical objectives set.

The Dimension VISTA 1500 instrumentation (Siemens, Munich, Germany) was used to dose creatinine (v.n. 0.6–1.17 mg/dL) using the enzymatic method, and serum calcium (v.n. 8.5–10.6 mg/dL) and phosphorus (v.n. 3.1–5.90 mg/dL) using the colorimetric method.

### 2.6. Statistical Analyses

The authors used a posteriori approach, as exclusion criteria were applied after samples were collected from all respondents. Statistical power analysis was performed to verify the suitability of the sample size of evaluated subjects (H vs. P). A significance > 80% was considered suitable.

A summary statistics of all subjects (H, P, P1 and P2) of the concentrations of biomarkers of bone metabolism and 25OHD 25(OH)D was provided, reporting means, medians, distribution at the 95% confidence interval (CI), and evaluation of the normality of the distribution (D’Agostino–Pearson test). A *p* value < 0.05 was considered statistically significant.

In H subjects, Tukey’s test was used to identify and exclude any anomalous data in the concentration of biomarkers. The presence of even a single value of a bone metabolism biomarker “suspected outliers” or 25OHD 25(OH)D deficiency status based on circulating levels < 20 ng/mL (<50 nmol/L) was used as a criterion to exclude normal subjects from the statistical calculation.

Boxplots of the distribution of bone biomarker values and 25OHD 25(OH)D were used to verify the correct selection of data included in the statistical evaluation in normal subjects (H) and to illustrate the different concentrations among normal subjects with PIC. The non-parametric Mann–Whitney U test was used to evaluate the differences in median concentrations among the different groups H, P, P1, and P2. Variables with a significance level lower than 5% were considered statistically significant.

Receiver operating characteristic (ROC) curves were used to evaluate the true positive rate (sensitivity) versus the false positive rate (specificity 100%) and were used to identify the concentration for each analyte where the best sensitivity and specificity were found.

The area under the ROC curve (AUC) provided a measure of the ability of each of the analytes included in the study to distinguish between the healthy group (H) and the total ICP group (P) and subgroups P1 and P2. The AUC was rated “excellent” for values between 0.9 and 1; “very good” between 0.8 and 0.9; “good” between 0.7 and 0.8; “fair” between 0.6 and 0.7; “sufficient” between 0.5 and 0.6; and “poor” and <0.5 (useless test) [34].

For statistical analysis, the MedCalc software program, version 11.6.1.0 (MedCalcSoftware, Mariakerke, Belgium), and Analite.it were used.

## 3. Results

Sample size power analysis performed using G*Power software (ver. 3.1.9.7; Heinrich-Heine-Universität Düsseldorf, Düsseldorf, Germany) indicated that a sample size of 56 subjects provided a significance level of greater than 80% for BALP and 95% for 25OHD, CTX, and OC.

The tooth vitality test resulted negative in all selected samples.

The summary statistics of all subjects (H, P, P1, and P2), bone metabolism biomarker concentrations, and 25OHD 25(OH)D are reported in Table 1. The distribution of biomarker values and 250HD 25(OH)D was normal (*p* < 0.05; D’Agostino–Pearson test) in the H sample group and is reported in Table 1. The lowest and highest 25OHD 25(OH)D concentrations in serum of normal subjects (H) were 22 and 34 (ng/mL), while for the P groups, they were 7 and 21 (ng/mL); for the P1 group, they were 10 and 21 (ng/mL); and for the P2 group, they were 7 and 11 (ng/mL). All apparently healthy subjects (H) and patients with cysts (P) included in the study did not present biochemical indices of systemic inflammation.

**Figure 2:**
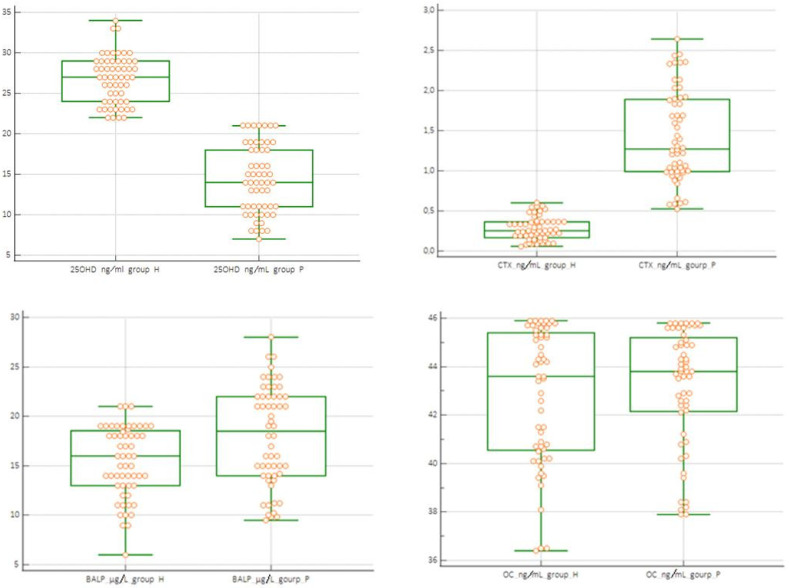
Box-plots of the distribution of the analytes dosed in H (healthy group) and P (pathological group) groups.

**Figure 3:**
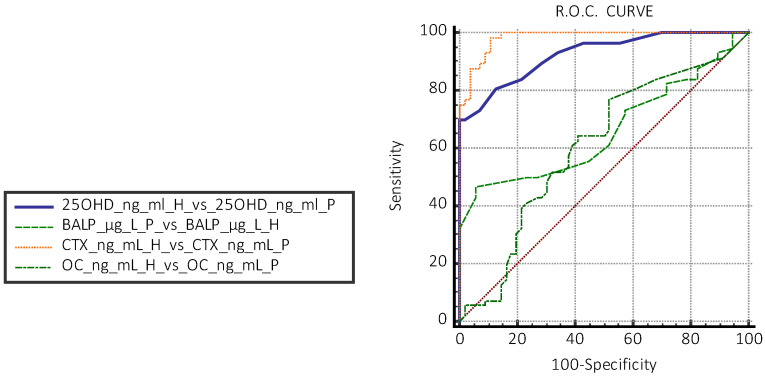
Evaluation of ROC curves between apparently healthy subjects (H) vs. patients with cysts (P) of 25OHD 25(OH)D, CTX, BALP, and OC (H = healthy group, P = pathological group).

In the H group, the evaluation with the Tukey test did not highlight the presence of aberrant data of the concentrations of CTX, BALP, OC, and 250HD 25(OH)D. The results obtained are reported graphically in Figure 1 and Figure 2, which display the boxplot of the concentration of BALP (ug/L) CTX (ng/mL), OC (ng/mL), and 25OHD 25(OH)D (ng/mL), obtained from the normal subjects (H) and in patients with PIC (P) evaluated. The graphs do not highlight the presence of values of the biomarkers of bone metabolism “suspected outliers” or concentrations of 25OHD 25(OH)D that identify a deficiency state [<20 ng/mL (<50 nmol/L)] in the normal subjects group (H). The box-plots analysis highlights differences in the distribution of the values of 25OHD 25(OH)D and CTX between the H group and the P group (Figure 2). The non-parametric Mann–Whitney U test showed a statistically significant difference between the median concentrations of 25OHD 25(OH)D and CTX of the subjects in the H group versus those in the P group (*p* < 0.05). The differences were not statistically significant in the groups of subjects evaluated between the concentrations of BALP and OC (Table 2a). Based on the results obtained, the significance of the median concentrations of the two analytes 25OHD 25(OH)D and CTX in the subgroups H vs. P1 and H vs. P2 and between P1 vs. P2 was verified. The test confirmed the significance of the differences in the median concentrations of 25OHD 25(OH)D and CTX, regardless of the number of cysts reported (Table 2b). The study of the ROC curves with a comparison between concentrations in the H vs. P group showed the best sensitivity and specificity for 25OHD 25(OH)D at a concentration < 19 ng/mL; this concentration is lower than that indicated as sufficient by the guidelines [35] for CTX-1 at a concentration > 0.561 ng/mL for BALP at a concentration > 19 μg/l., and for OC at a concentration < 41.5 ng/mL (Table 3). The AUCs of 25OHD 25(OH)D and CTX equal to 0.92 and 0.96, respectively, were evaluated as “excellent”, while the AUCs of BALP and OC had “sufficient” accuracies of 0.66 and 0.51, respectively (Figure 3).

## 4. Discussion

Periapical lesions and in particular PIC are the consequence of a dynamic encounter between microbial factors present in the root canals and host defence mechanisms with the appearance of local inflammation, bone resorption, and destruction of other periapical tissues. Cysts are among the most frequently diagnosed odontogenic pathologies in the oral cavity with the production of cytokines that also activate osteoclastogenesis.

In clinical practice, the diagnostic approach and follow-up of these pathologies involves the use of radiological and/or clinical examinations. The therapy is essentially surgical. In our study, 16 cystic lesions were treated endodontically with resolution, and 40 cysts were treated with cystectomy, of which 21 were associated with apicectomy after failure of endodontic treatment of the affected teeth and 19 with extractions of the compromised teeth.

In 21 subjects with apical lesion due to failed endodontic treatment (presence of inadequately sealed canals), apicectomy was performed with resolution of the lesion at 6-month follow-up. Histological examination of the removed lesions confirmed the diagnosis of PIC.

The mechanism of PIC growth depends on cellular activity stimulated by inflammatory mediators, which activate fibroblasts with the release of collagenase and prostaglandins and activate osteoclasts with subsequent bone resorption.

Biochemical markers for the evaluation of bone remodelling during both acute and chronic inflammatory processes of the stomatognathic system have been studied [18]. In particular, the concentrations of TRAcP-5, RANKL, and OPG during symptomatic and asymptomatic periapical cysts have been studied both at the serum and salivary levels; of CTX, OC, and BALP during periodontal diseases and of calprotectin and N-terminal telopeptide (NTx) in patients suffering from peri-implantitis; and of OC, deoxypyridinoline, CTX, N-terminal telopeptides, BALP, and parathyroid hormone in patients suffering from osteonecrosis of the jaw bones related to bisphosphonates. The preliminary results of our study have highlighted that in subjects with both single and multiple periapical cysts, there is an increase in CTX concentration with a statistically significant difference between the groups of subjects evaluated (H vs. P1 and P2).

CTX is a marker of bone resorption; in fact, it is produced during bone degradation processes, and its serum levels are significantly correlated with bone density measurements. The prevalence of resorption processes compared to apposition phenomena is demonstrated by the absence of statistically significant variations in the OC and BALP markers.

Based on the results on the concentration of biomarkers, it can be hypothesised that for the formation of periapical cysts, an osteoclastic activity unbalanced towards local bone resorption is necessary. This process is certainly correlated to the immunoinflammatory response to endodontic microbial infection and by a continuous cytokine-mediated immune response with involvement of T lymphocytes, which intervene in both humoral and cell-mediated immunity.

25OHD is essential for calcium metabolism, bone turnover, and immune regulation, and it has anti-inflammatory effects. 25OHD also has protective effects against a myriad of chronic diseases and infectious diseases. A deficiency of 25OHD is associated with accelerated bone turnover, reduced bone density, and increased risk of bone fractures. Vitamin D deficiency can determine an increase in alveolar bone loss and gingival inflammation in predisposed subjects. Poor nutrition and inadequate 25OHD status affect oral health and function. Human gingival cells are able to produce 25-hydroxylase, which is responsible for the production of 25OHD and explains the production of vitamin D within the periodontium.

25OHD binds to immune cells (monocytes, macrophages, dendritic cells) to enhance chemotactic and phagocytic activities, and to junctional and gingival epithelial cells, participating in epithelial defence mechanisms against invading pathogens. Administration of 25OHD reduces inflammation by suppressing the expression of receptor activator of nuclear factor kappa beta ligand (RANKL), TNF-α, IL-1, and IL-6, as well as a reduction in alveolar bone loss.

It also appears that the impact of 25OHD status on periodontal disease might be more pronounced in some vulnerable patient groups (menopausal or pregnant women or elderly subjects).

Ilan Rotstein observed that the presence of periapical abscesses is higher in patients with 25OHD deficiency than in patients without 25OHD deficiency and that the administration of 25OHD could reduce the risk of the onset of this pathology in 25OHD-deficient patients.

Therefore, studies suggest that total 25OHD intake is inversely associated with the probability of severe periodontal disease. Mechanisms of osteoblastic activation with immunoinflammatory response to microbial infection for the formation of PIC can be interpreted on the basis of the detection of 25OHD concentration values below the state considered sufficient (20 ng/mL) by various international guidelines; in fact, most scientific societies have defined as “deficient” a 25OHD level < 10 ng/mL, “insufficient” if <20 ng/mL, and “optimal” if between 20 and 50 ng/Ml.

Our ROC curve study comparing concentrations in the H vs. P group showed a discriminant cut-off for a concentration < 19 ng/mL, severe insufficiency, and a statistically significant difference between median 25OHD concentrations. If clinical studies have consistently demonstrated an inverse relationship between 25OHD inflammation and periodontal disease, the involvement of 25OHD in the pathogenesis of PIC is biologically plausible.

Severe 25OHD insufficiency could favour the onset and evolution of PIC, intervening in the modulation of antimicrobial immune responses, the production of inflammatory cytokines and bone mineralisation, with a prevalence of resorption phenomena compared to those of apposition, as highlighted by us from the levels of CTX vs. OC and BALP. The diagnosis of PIC, like other chronic inflammatory disease of the oral cavity, may be accompanied by a deficiency of 25OH (<20 mg/dL) and therefore provides an indication for initial monitoring.

The Endocrine Society guidelines do not recommend the determination of 25OH as a screening test to determine the state of hypovitaminosis in the asymptomatic population for possible supplementation.

Other scientific societies have also stated that it is not necessary to perform extensive screening of 25OHD without clinical signs.

In truth, the US National Academy of Medicine has set the adequacy threshold at 50 nmol/L equal to 20 mg/dL, and there is a growing consensus about levels at or above the threshold of 75 nmol/L equal to 30 mg/dL.

The presence of apical cysts could instead be a clinical condition for a clinically appropriate dosage.

PIC should be recognised by dental specialists as a clinical condition which can be accompanied with potential 25OHD deficiency, especially in the presence of subjects at risk of osteopenia (menopause, pregnancy, aging). Furthermore, it would be appropriate to evaluate whether 25OHD supplementation in deficient patients could determine benefits in preventing the progression of PIC disease.

PIC are more than half of odontogenic cysts, and they appear between the ages of 20 and 60, both in males and females, with a predominantly anterior maxillary location (60%) followed by the posterior maxillary, posterior mandibular, and anterior mandibular portions, with dimensions ranging from a few millimetres to a few centimetres [5]. They are most commonly found at the apices of necrotic teeth or canals with defective root canal fillings [36]. PIC originate from epithelial residues (Malassez remnants) present in apical granulomas stimulated by cytokines produced by inflammation in an attempt to separate the inflammatory stimulus from the surrounding bone [37]. They are the consequence of a dynamic encounter between microbial factors present in the root canals and host defence mechanisms with the appearance of local inflammation, bone resorption, and destruction of other periapical tissues [38,39].

In clinical practice, the diagnostic approach and follow-up of these pathologies involve the use of radiological and/or clinical examinations, and the therapy is essentially surgical. In this study, 16 cystic lesions were treated endodontically with resolution; 40 cysts were treated with cystectomy, of which 21 were associated with apicectomy after failed endodontic treatment of the affected teeth; and 19 were treated with extractions of the compromised teeth. In 21 subjects with apical lesion due to failed endodontic treatment (presence of inadequately sealed canals), apicectomy was performed with resolution. All patients were followed up for 6 months, and in no patient was there complications or recurrences. Histological examination of all removed lesions confirmed the diagnosis of PIC.

The growth mechanism of PIC depends on cellular activity stimulated by inflammatory mediators, which activate fibroblasts with the release of collagenase and prostaglandins and activate osteoclasts with subsequent bone resorption [18]. The presence of necrotic tissue and bacterial endotoxin causes an inflammatory response in the periapical area. It has been reported that bacterial endotoxin directly stimulates the proliferation of keratinocytes and inflammatory response cells (fibroblasts, granulocytes, macrophages, and lymphocytes), with the release of a series of cytokines (prostaglandin (PG), interleukin (IL), interferon (IFN), tumour necrosis factor alpha (TNF-α), and growth factors), capable of modulating the immune response [4,5].

Proinflammatory cytokines, interleukins, prostaglandins, and TNF-α are known to stimulate bone resorption through upregulation of the NFβ ligand (RANKL), which belongs to the TNF-α super family [40]. The actions of RANKL include promoting osteoclast differentiation, inducing osteoclast activation, survival, and adherence to the bone surface [41].

Alveolar bone resorption by osteoclasts is the dominant destructive event in radicular cysts. In fact, the differentiation of osteoclast precursor cells into mature osteoclasts is responsible for the subsequent bone resorption and is stimulated by RANKL, produced by osteoblasts and osteocytes as well as by T lymphocytes and monocytes [36,42].

In radicular cysts, it is possible to identify the presence of RANKL and its physiological inhibitor osteoprotegerin (OPG), with a strongly positive RANKL/OPG ratio (1.40 ± 0.04) and an indicator of bone resorption [40,41,43,44].

Recently, it has been suggested that the RANKL/RANK/OPG system is essential not only for bone biology but also for the activation of immune functions. RANKL binding to its receptor, RANK, is present on the surface of pre-osteoclasts and immune response cells [44], allowing the regulation of bone metabolism and a correct functioning of the immune system [45].

It has been reported that in radicular cysts, cytokines produced by inflammatory cells can

- promote alveolar bone loss by activating an excessive pro-inflammatory response and reversing the OPG/RANKL ratio [46];
- stimulate the proliferation of osteoclasts, causing a further stimulus to the alveolar bone resorption process [47,48];
- activate T lymphocytes capable of producing cytokines that modulate the nature of the host immune response [49].

This process of T-cell activation is strongly influenced by vitamin D [50].

Vitamin D is a steroid hormone that plays an important role in immune regulation and anti-inflammatory activity [51]. It regulates calcium–phosphate homeostasis and bone metabolism and therefore may influence alveolar bone mineral density [52,53].

A deficiency of 25(OH)D is associated with accelerated bone turnover and reduced bone density [51].

Numerous observations suggest that maintaining adequate vitamin D levels, i.e., between 30 and 80 ng/mL (75–200 nmol/L), is important for maintaining immune system function [54].

Vitamin D has been shown to be essential for normal macrophage function, and its deficiency has been linked to impaired chemotaxis; phagocytosis [8]; and upregulation of monocyte toll-like receptors, known inducers of inflammation [55]. In vivo and in vitro experiments have demonstrated that vitamin D modulates the production of anti-inflammatory cytokines (transforming growth factor (TGF β-1) and IL-4) and reduces the production of pro-inflammatory ones (IL-6, IFN-γ, IL-2, and TNF-α) [56,57].

Increased cytokine expression associated with the inflammatory response underlies the interaction between vitamin D and RANKL. It is known that there are overlapping regulatory mechanisms between the immune system and the bone system; cytokines produced by lymphocytes and macrophages act as important promoters of osteoimmunological regulation [58].

Furthermore, vitamin D exerts a potent antimicrobial effect against periodontal pathogens by directly inhibiting bacterial growth [59], facilitating the production of antimicrobial peptides such as β-defensin and cathelicidin [60] by increasing the binding of junctional and gingival epithelial cells, which participate in epithelial defence mechanisms against invading pathogens [61].

Rotstein I et al. observed that the presence of periapical abscesses is higher in patients with 25(OH)D deficiency than in patients without 25(OH)D deficiency and that vitamin D supplementation may reduce the risk of developing this disease in patients with 25(OH)D deficiency [39]. Furthermore, prospective, association, and retrospective studies have demonstrated that prenatal maternal vitamin D deficiency (serum 25(OH)D concentrations < 40 ng/mL) is associated with an increased risk of deciduous tooth caries and enamel hypoplasia [62,63].

There is considerable evidence that poor nutrition and inadequate vitamin D status can influence oral health and function [3]. Vitamin D deficiency can lead to increased alveolar bone loss and predispose to gingival inflammation, especially in predisposed subjects [64,65].

In fact, there are several studies that have highlighted a correlation between the degree of periodontal health and 25(OH)D deficiency.

The National Health and Nutrition Examination Survey (NHANES) reports that serum vitamin D levels are negatively associated with periodontal disease and the severity of the clinical picture [66,67]; increased vitamin D levels reduce gingival inflammation, and the anti-inflammatory effect of vitamin D is dose-dependent [68].

The importance of vitamin D for the oral cavity is also evident from the fact that, to increase the effectiveness of the vitamin’s action, human gingival cells are able to produce 25-hydroxylase, responsible for the production of active vitamin D within the periodontium [69].

This is the first study that has correlated the presence of PIC with the state of 25(OH)D deficiency.

In this observational study, a statistically significant difference was found between the median concentrations of 25(OH)D of the subjects in the apparently healthy group compared to those with cysts (25(OH)D < 19 ng/mL) (*p* < 0.05) and a significant reduction in the level from “insufficient” to “deficient” as a function of the number of cysts (25 OHD < 11 ng/mL).

A cut-off of 19 ng/mL was found to be highly discriminating for the concentrations in the cyst group compared to the apparently healthy group. That is, vitamin D concentrations indicating “severe insufficiency” identified subjects with PIC with high sensitivity and specificity [35].

Severe 25(OH)D deficiency (<20 ng/mL) could favour the onset and evolution of PIC, as a consequence of an insufficient antimicrobial immune response and bone resorption phenomena.

Vitamin D deficiency favours bone resorption phenomena, and this process is observed in the course of PIC; therefore, in this study, some biomarkers of bone metabolism were evaluated: CTX, OC, and BALP. The results showed that in subjects with single and multiple periapical cysts an increase in the concentration of CTX, a marker of bone resorption, was observed, with a statistically significant difference between the apparently healthy group and the group of patients with PIC, regardless of the number of cysts reported.

CTX concentrations higher than 0.561 ng/mL were suitable for differentiating the group of apparently healthy subjects from the group of patients with PIC, with “excellent” accuracy (AUC equal to 0.96); the cutoff value was higher than the median of 0.250 ng/mL of a reference interval stratified by adult population and geographical location [70]. The prevalence of resorption processes compared to apposition phenomena is demonstrated by the absence of statistically significant variations in the OC and BALP markers.

Based on the results obtained, it can be hypothesised that the formation of periapical cysts presents an osteoclastic activity unbalanced towards “local” bone resorption. This process is certainly related to the immuno-inflammatory response; to endodontic microbial infection; and to a continuous immune response mediated by cytokines with the involvement of T lymphocytes, which intervene in both humoral and cell-mediated immunity [3]. The detection of apical cysts could be a suitable clinical condition for an appropriate dosage of vitamin D, useful for determining the state of hypovitaminosis, especially in subjects at risk of osteopenia [71,72].

### Limit of Study

This study has limitations related to the number of patients enrolled. This number is not large enough for the very restrictive inclusion criteria. Further clinical and experimental validations with broader statistical analysis and larger samples will be needed to confirm the hypothesis of this pilot study.

## 5. Conclusions

This pilot study is the first that investigated 25OHD, CTX, OC, and BALP levels in patients with PIC. The statistically significant variations in CTX concentration compared to OC and BALP highlighted the prevalence of resorption processes compared to apposition phenomena in subjects with PIC. The severe 25OHD insufficiency could favour the onset and evolution of PIC by acting on the chronic inflammatory process and resorption processes. PIC could be accompanied to be considered a sign of 25OHD deficiency; therefore, it would be desirable to investigate the levels of serum 25OHD in the presence of these oral lesions. Further clinical and experimental validations with broader statistical analysis and larger samples will be needed to confirm the hypothesis of this pilot study.

This pilot study is the first to investigate 25(OH)D, CTX, OC, and BALP levels in patients with ICP. Statistically significant changes in CTX concentration are a biochemical sign of the prevalence of reabsorption processes over apposition phenomena in subjects with PIC. The severe 25(OH)D deficiency found in these patients could favour the onset and evolution of PIC by acting on the chronic inflammatory process and on the reabsorption processes. PIC could be considered a sign of vitamin D deficiency and therefore provide a recommendation to investigate serum 25(OH)D levels.

## Figures and Tables

**Table 1:** Characteristics of the sample (H = healthy group, P = pathological group; P1 = patient with only one PIC; P2 = patient with two or more PIC).

	25OHD ng/mL GroupH	25OHD ng/mL Group P	25OHD ng/mL Group P1	25OHD ng/mL Group P2	BALP µg/L Group H	BALP µg/L Group P	BALP µg/L Group P1	BALP µg/L Group P2	CTX ng/mL Group H	CTX ng/mL Group_P	CTX ng/mL Group P1	CTX ng/mL Group P2	OC ng/mL Group_H	OC ng/mL Group P	OC ng/mL Group P1	OC ng/mL Group P2
N	56	56	42	14	56	56	42	14	56	56	42	14	56	56	42	14
Minimum	22	7	10	7	6	9.5	9.5	9.8	0.059	0.526	0.526	1.541	36.40	37.90	37.90	43.80
Maximum	34	21	21	11	21	28	26	28	0.60	2.64	2.34	2.64	45.9	45.8	45.7	45.8
Mean	26.7	14.32	15.9	9.214	15.39	18.02	17.58	19.34	0.278	1.423	1.199	2.097	42.83	43.18	42.50	45.43
95% CI	25.9 to 27.5	13.19 to 15.8	14.93 to 17.02	8.49 to 9.93	14.44 to 16.34	16.67 to 19.37	16.13 to 19.03	15.85 to 22.83	0.24 to 0.31	1.27 to 1.57	1.06 to 1.33	1.89 to 2.29	42.07 to 43.58	42.53 to 43.84	41.74 to 43.26	45.09 to 45.77
Median	27	14.0	15.5	9.5	16	18.5	16.5	21.5	0.254	1.27	1.07	2.09	43.6	43.8	43.20	45.75
95% CI	26 to 28	13 to 15	14.18 to 18.0	8. to 10	14 to 18	15 to 21	15 to 20.81	13.60 to 23.00	0.22 to 0.33	1.07 to 1.62	0.99 to 1.25	1.85 to 2.36	41.50 to 44.74	42.90 to 44.46	42.23 to 43.88	44.99 to 45.80
SD	2.95	4.2	3.35	1.25	3.56	5.03	4.655	6.045	0.142	0.571	0.438	0.349	2.816	2.454	2.436	0.587
RSD	0.11	0.56	0.20	0.13	0.23	0.27	0.264	0.312	0.513	0.401	0.366	0.166	0.065	0.056	0.057	0.012
5-95 P	21.0 to 32.1	7.00 to 21.0	11.00 to 19	7.20 to 11.00	9.30 to 20.40	9.86 to 25.7	10.6 to 24.4	9.80 to 27.6	0.08 to 0.55	0.58 to 2.41	0.58 to 2.00	1.55 to 2.60	36.9 to 45.9	38.1 to 45.8	38.0 to 45.6	44.0 to 45.8
Normal Distr.	0.65	0.003	0.003	0.503	0.207	0.003	0.015	0.426	0.172	0.047	0.139	0.505	0.058	0.028	0.083	0.009
	Accept normal	Reject norma	Accept normal	Accept normal	Accept normal	Reject normal	Reject norma	Accept normal	Accept normal	Reject normal	Accept normal	Accept normal	Accept normal	Reject normal	Accept normal	Reject normal

**Table 2:** (**a**) Mann–Whitney test of 25OHD 25(OH)D, CTX, and BALP E O in H vs. P, H vs. P1, H vs. P2, and P1 vs. P2. (**b**) Mann–Whitney test of 25OHD 25(OH)D and CTX in sottogruppo subgroups H vs. P1, H vs. P2, and P1 vs. P2.

(a)		
Comparison Sample	Mann–Whitney Test	* p * Value
Group H vs. P		
25OHD	236	<0.001
CTX	7	<0.001
BALP	1253	>0.05
OC	1235	>0.05
**(b)**		
** Comparison Sample **	** Mann–Whitney Test **	*** p *** ** Value **
Group H vs. P1		
25OHD	236	<0.001
CTX	7.01	<0.001
Group H vs. P2		
25OHD	2.5	<0.001
CTX	0.01	<0.001
Group P1 vs. P2		
25OHD	9.5	<0.001
CTX	40	<0.001

H = healthy group, P = pathological group; P1 = patient with only one PIC; P2 = patient with two or more PIC.

**Table 3:** The AUC values of the parameters taken into consideration in the H vs. P.

Group H vs. P	Dosage	AUC	Sensitivity %	Specificity %
25OHD	≤19 ng/mL	0.925	91	99
CTX	0.561 ng/mL	0.96	92	98
BALP	>19 µg/L	0.660	46	94
OC	41.5 ng/mL	0.512	76	39

H = healthy group, P = pathological group.

## Data Availability

The data presented in this study are available from the corresponding author upon reasonable request.
